# Editorial: Endocrinology of obesity, aging and stress

**DOI:** 10.3389/fendo.2025.1587811

**Published:** 2025-03-27

**Authors:** Yuko Maejima, Kenju Shimomura, Heidi de Wet

**Affiliations:** ^1^ Department of Bioregulation and Pharmacological Medicine, Fukushima Medical University, Fukushima, Japan; ^2^ Department of Physiology, Anatomy and Genetics, University of Oxford, Oxford, United Kingdom

**Keywords:** aging, obesity, stress, endocrinology, metabolism

The intricate relationship between the endocrine system, obesity, aging, and stress has become a focal point in contemporary research. This Research Topic, “*Endocrinology of obesity, aging, and stress*,” gathers pivotal studies that collectively enhance our understanding of these interrelated physiological systems. By examining the fields of hormonal, metabolic, and psychological systems, these contributions offer a comprehensive and integrated view of the factors influencing health and disease in the context of obesity, aging, and stress.

Various studies within this Research Topic have illuminated the significant roles of body composition and metabolic health in aging processes, highlighting how certain body indices can serve as predictors of important anti-aging markers. This perspective is further enriched by research into the regulatory mechanisms of body composition, emphasizing the therapeutic potential of targeting specific genetic markers and metabolic pathways to manage health. Global research trends on aging associated with periodontal health over the last two decades reveal significant technical advancements, providing a rich backdrop for future research directions in this field. The exploration of psychological factors also proves crucial, as emotional and social support systems are shown to significantly impact glycemic control, particularly in patients managing chronic conditions such as diabetes over a lifetime. These studies highlight that the mental health aspect of dealing with long-term conditions needs as much attention as the management of the physiological pathologies associated with chronic disease.

Mechanisms underlying weight regulation have been another critical area of focus. Investigations into hormonal influences reveal that processes beyond traditional sympathetic innervation contribute to the regulation of body weight and adiposity, underscoring the complexity of endocrine interactions. Moreover, novel therapeutic agents have been identified that may offer new avenues for obesity treatment, acting on specific hormonal receptors to reduce energy intake and promote weight loss. Additionally, comprehensive assessments of body composition beyond BMI are emphasized for evaluating health risks, particularly in the context of obesity-related mortality. The predictive value of obesity and lipid-related indices in the onset of type 2 diabetes underscores the importance of regular monitoring and early intervention to mitigate health risks.

The relationship between metabolic health and cognitive function, particularly in aging populations, is another vital aspect explored in this Research Topic. Findings suggest a clear link between metabolic indices and cognitive performance, highlighting the importance of maintaining metabolic health to preserve cognitive abilities in older adults. Early detection and intervention strategies based on advanced metabolic profiling techniques offer promising pathways to mitigate risks associated with muscle deterioration and cognitive decline.

Gender-specific responses to stress and therapeutic interventions are also addressed, with research indicating that aromatherapy and other non-pharmacological treatments may have differing effects based on sex. This highlights the necessity for personalized approaches in stress management and therapeutic applications. Reproductive health factors, such as age at first birth and menopause, are shown to influence the risk of ovarian cysts, offering insights into the long-term impacts of reproductive milestones.

Clinical interventions for degenerative diseases muscle degeneration and cervical disc degenerative diseases are also examined, providing guidance on optimal surgical approaches to improve patient outcomes.

The long-term outcomes and potential complications of surgical treatments for benign prostatic hyperplasia, as well as the significant impact of abdominal obesity on mortality rates in specific populations, highlight the need for targeted interventions to improve survival rates and patient care.

In conclusion, the articles featured in this Research Topic collectively advance our understanding of the endocrine, metabolic, and psychological factors influencing obesity, aging, and stress. By integrating diverse research methodologies and interdisciplinary approaches, these studies provide a robust foundation for future exploration and innovation in health promotion and disease prevention strategies.

